# Supplementary material for: Patterns of Intron Gain and Loss in Fungi
Source: PLoS Biol. 2004 Nov 30;2(12):e422. doi: 10.1371/journal.pbio.0020422 (PMC532390; doi:10.1371/journal.pbio.0020422)
Supplement: Table S1 — Also available at http://genes.mit.edu/NielsenEtAl/. (4.3 MB ZIP). [file pbio.0020422.st001.zip › NielsenEtAl/html/456.html]

AN1334.1.NCU03883.1.MG01006.1.FG09952.1


```
 CLUSTAL W (1.82) Multiple Sequence Alignments - Introns Inserted


Sequence 1: NCU03883.1	127 aa
Sequence 2: FG09952.1	129 aa
Sequence 3: MG01006.1	128 aa
Sequence 4: AN1334.1	123 aa
Alignment Length: 129 aa
Number Identitical Residues: 88 aa
Alignment Score (without introns) 3742


MG01006.1 	MSSQPLLQTAPG1KRIALPTRVEPKVFFANERTFLSWLNFTVVLGALAVGMLNFGDRPAF
NCU03883.1	MSSQPLLQTAPG1KRIALPTRVEPKVFFANERTFLSWLNFTVILGALAIGMLNFGDRPAF
FG09952.1 	MSSQPLLQTTPG1KRIALPTRVEPKVFFANERTFLSWLNFTVILGALAIGMLNFGDRVAF
AN1334.1  	MSSQPLLQTAPG1KRIALPTRVEPKVFFANERTFLSWLNFTVILGGLAVGLLNFGDRVGR
          	*********:** *****************************:**.**:*:****** . 

MG01006.1 	ISSIMFTLVAMFTMGYALWQYHWRAATIRKRGQAGFDDRFGPTMLAIILFLAVVVNFVLR
NCU03883.1	ISAFLFTGVAMATMVYALVTYHWRAKSIRLRGQAGFDDRFGPTFLALILLLAVVVNFVLR
FG09952.1 	ISAFLFTGVAMCTMVYALVTYHWRAQSIRMKGQAGFDDRFGPTVLAIILLLAVVVNFVLR
AN1334.1  	ISAGLFTVIAMAAMIYALVTFHWRAQSIRKRGHSGIDDRFGPTILAISLLAAVVVNFVLR
          	**: :** :** :* ***  :**** :** :*::*:*******.**: *: *********

MG01006.1 	VTYDKNKKV-
NCU03883.1	ITYISQQP--
FG09952.1 	ITDKSKNHSN
AN1334.1  	MLQD------
          	:
```
